# Identifying indoor radon sources in Pa Miang, Chiang Mai, Thailand

**DOI:** 10.1038/s41598-020-74721-6

**Published:** 2020-10-20

**Authors:** Tarika Thumvijit, Supitcha Chanyotha, Sompong Sriburee, Pongsiri Hongsriti, Monruedee Tapanya, Chutima Kranrod, Shinji Tokonami

**Affiliations:** 1 Department of Radiologic Technology, Faculty of Associated Medical Sciences, Chiang Mai University, Chiang Mai, 50200 Thailand; 2 Center of Radiation Research and Medical Imaging, Chiang Mai University, Chiang Mai, 50200 Thailand; 3 Department of Nuclear Engineering, Faculty of Engineering, Chulalongkorn University, Bangkok, 10330 Thailand; 4 Natural Radiation Survey and Analysis Research Unit, Chulalongkorn University, Bangkok, 10330 Thailand; 5 Department of Radiation Measurement and Physical Dosimetry, Institution of Radiation Emergency Medicine, Hirosaki University, Hirosaki, Aomori 036-8564 Japan

**Keywords:** Environmental sciences, Health occupations

## Abstract

Radon is the leading source of lung cancer mortality after smoking in Chiang Mai, Thailand. Finding a source of carcinogens is one of the important measures for preventing the cancer risk for this region. Specific sites at Pa Miang, Doi Saket have the highest incidences of lung cancer and have a combination of factors that influence indoor radon concentration. Our study identified the sources of indoor radon within several houses. The results indicate that geological and topographic characteristics, including active faults and mountain terraces, are the main sources of indoor radon, especially for wooden houses. Besides building materials, the design of the houses, ventilation conditions, and lifestyle choices are all factors influencing indoor radon concentrations and its associated risk. Although radon levels (29–101 Bq m^−3^) and total indoor annual effective doses (0.9–3.8 mSv year^−1^) received from all sources at these sites have shown no significant health risk due to radon exposure , this investigation will be useful as a starting point to guide strategies to respond and prevent the risk of lung cancer, especially in Chiang Mai.

## Introduction

Humans are naturally exposed to different levels of radioactivity, depending on the radioactivity that is naturally present in the area and the environment associated with geological features, particularly thorium, uranium and potassium in rocks from which the soil originated. Radon (^222^Rn) is a natural radioactive noble gas that occurs in the uranium decay series and is formed directly from the alpha decay of radium (^226^Ra). Since radon is an inert radioactive gas, it does not react chemically with other elements and tissues and prefers to stay in a gas phase instead of dissolving in water. Radon has a half-life of 3.82 days, making it the largest component (42%) of the average radiological exposure dose to the general public as reported by UNEP^1^.

UNEP^1^ estimated that the average annual effective dose (AED) of radiation to the public is 3 mSv, with natural sources contributing about 2.4 mSv of which two thirds comes from radioactive substances in the air we breathe (1.3 mSv) and the food and water we ingest (0.3 mSv)^2^. Radon can enter the human body in two different ways, by inhalation through the respiratory tract and by ingestion via the gastrointestinal tract. Both mechanisms constitute potential health hazards. When inhaled, some of radon’s short-lived decay products (mainly polonium-218 and -214) are retained in the lungs and irradiate cells in the respiratory tract with alpha-particles. Radon is, hence, a primary contributor to an increased risk of lung cancer and a cause of lung cancer in both smokers and non-smokers. The National Research Council^3,4^ reported that breathing radon in indoor air is the second largest contributor to lung cancer after smoking. Several studies have reported that patients have died from lung cancer due to inhalation of radon^2,4–13^. The recent ICRP publication 126^14^ recommends long-term average indoor Rn concentration 300 Bq m^−3^ as upper limit of the choice of national reference level. This corresponds to annual effective dose of 4 mSv at work and 14 mSv at home^15^.

However, since radon is somewhat soluble in water, if a high concentration is found in drinking water, this can be a significant route of intake. This could be particularly true in groundwater, since radon activity concentrations there are often several orders of magnitude higher than in surface waters^16^. Of course, radon in water can be a pathway of entry into homes and other structures where it becomes available for respiration by degassing. The World Health Organization^17^ estimated that 1–7% of all lung cancer deaths are due to high levels of radon in water and that 10–15% of total indoor radon levels may be attributed directly to out-gassing from tap water. Based on a National Academy of Science report^18^, the EPA estimates that in the USA, radon in drinking water causes about 168 cancer deaths per year: 89% from lung cancer caused by breathing radon released to the indoor air from water and 11% from stomach cancer caused by consuming water containing a high level of radon. Nevertheless, a review of the international research data^2^ concluded that on average, 90% of the dose attributable to radon in drinking water comes from inhalation rather than ingestion. In other words, the health risks associated with the consumption of radon in drinking water are considered insignificant compared to the risks resulting from the inhalation of radon released from the water into the air.

Drinking-water is a fundamental requirement for life, and good quality drinking water is important to human health. The quality of drinking water depends on many factors, such as the quality of the water source, treatment methods, and the container used to store the water^19^. To protect the health of citizens from poor quality drinking water, international and national safety agencies have established safe drinking water quality criteria, including radon levels. The United States Environmental Protection Agency^16^ recommended the Radon in Drinking Water Rule in the *Federal Register* on November 2, 1999 (64 FR 59246) where groundwater or mixed ground and surface water should have a maximum concentration level (MCL) of 11 Bq L^−1^ (300 pCi L^−1^) and an alternative (A)MCL of 148 Bq L^−1^ (4000 pCi L^−1^) if community water systems introduce a multimedia mitigation (MMM) program to address the radon risks in indoor air. The Council of the European Union^20^ set a lower parametric value of 100 Bq L^−1^ for radon in drinking water. If the activity is over 1000 Bq L^−1^, then remedial measures should be taken and justified on radiological protection grounds without compromising the water supply on a national or regional scale.

With respect to the WHO’s publications on the health risks of radon in drinking water, the *Guidelines for drinking‑water quality (GDWQ),* published during 2004–2008^6,21^, gave a guidance threshold of 100 Bq L^−1^ for radon in drinking-water supplies. At radon concentrations in drinking-water above 100 Bq L^−1^ certain controls should be implemented by appropriate treatments, such as air-stripping, aeration systems or, for small water supplies, activated carbon adsorption. However, in a more recent publication^22^, the WHO preferred not to provide a guidance level for radon in drinking water but recommended instead an individual dose criterion (IDC) of 0.1 mSv year^−1^. The IDC of 0.1 mSv is for the consumption of drinking water over the course of 1 year (assuming 2 L day^−1^) regardless of whether the radionuclides are naturally occurring or human-made. In practice, the IDC is translated in the GDWQ into two operational quantities, screening levels and guidance levels. This would represent a very low level of health risk from the prolonged exposure to radionuclides in drinking water. Drinking-water is a fundamental requirement of life and the risks of not having a drinking-water supply are likely to be much greater than consuming drinking-water that does not meet the IDC. Therefore, WHO mentioned in the publication that the IDC should not be interpreted as a limit above which drinking water is unsafe for consumption^22^.

In the recent ICRP Publication 137^15^, the dose coefficient for radon ingestion (6.9 × 10^–7^ mSv Bq^−1^) and inhalation (6.7 × 10^–6^ mSv/[Bq h m^−3^]) was suggested, which a new biokinetic model for systematic radon was used to calculate the committed effective doses (EDs) following the ingestion of radon. The different radon criteria in water and drinking water introduced by international organizations are summarized in Table 1.

**Table 1:** International radon criteria for drinking water.

Directive/recommendation	Activity concentration (Bq L^−1^)	References
EURATOM DWD (E-DWD)	100–1000 ^a^	Council of the European Union^19^
US-EPA MCL	11.1	United States Environmental Protection Agency^15^
US-EPA AMCL	148	NRC^17^, United States Environmental Protection Agency^15^

Directive/recommendation	Dose criterion	References
WHO ^b^	0.1 mSv year^−1^	WHO^21^

^a^> 1000 Bq L^−1^ remedial action without further consideration is justified in all EU countries.

^b^An individual dose criterion (IDC) for the consumption of drinking water over the course of 1 year.

In Thailand, lung cancer has ranked as the top new cancer among males in the Chiang Mai province since population-based registration began in 1983^23^. In 2001, epidemiological studies performed to find the relationship between indoor radon and lung cancer in Saraphi district, Chiang Mai, Thailand, reported that smoking was the main factor of causing lung cancer, followed by the levels of indoor radon^24^. The indoor radon values throughout Thailand in 2011 were reported to be in the range 1–1974 Bq m^−3^ with the highest levels found in northern Thailand, which were the highest levels among East Asian countries^25^. That study also reported that apart from smoking, the indoor radon level was the main risk factor for lung cancer in in that area. Likewise, it was recently reported that annual average indoor radon activity concentrations in Chiang Mai province ranged from 35 to 219 Bq m^−3^^26^, where the maximum value is 5.5-fold higher than the world average concentration of indoor radon of 40 Bq m^−3^^14^. In addition, a high level of environmental radon pollution was classified as a public health hazard concern in Chiang Mai Province^26^.

The main source of radon gas is soil, but as a gas, it can enter buildings in many ways; through cracks or holes in foundations or concrete or wood floors, resulting in enhanced concentration levels. Knowing relevant geological and anthropogenic information can help to prevent entry. To investigate these effects, specific areas in the small villages located in Pa Miang subdistrict, Doi Saket district, Chiang Mai, Thailand were chosen to identify indoor radon sources for residents and to estimate the health risks due to the radiological effects from radon. This specific location is located in a “high radon potential zone”^27^ and sits in a basin of granitic rock associated with active faults^28,29^, is surrounded by mountains, and is known as one of the highest incidences of lung cancer cases among the 25 districts of Chiang Mai^26^. Moreover, the water supplies to households in this area is pumped directly from nearby different resources, private wells, springs or streams, with and without adequate treatment. Hence, it is important to investigate radon sources from water and other sources and assess resulting risk.

**Figure 1:**
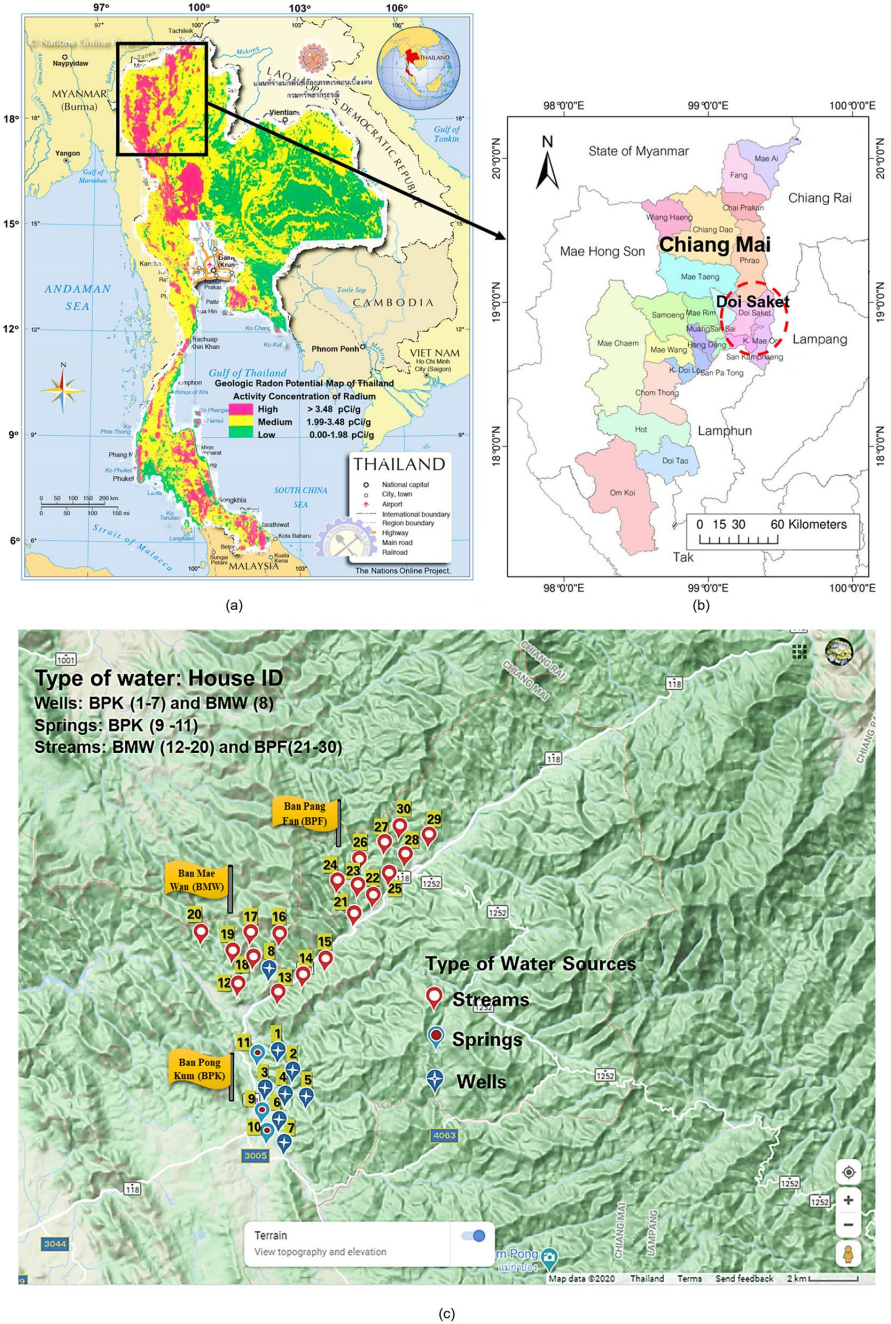
Study area and sample point locations. (**a**) Geologic radon potential map overlaid on the map of Thailand, (**b**) location of Doi Saket district in Chiang Mai Province, (**c**) sampling point locations, marked by a GPS instrument (Garmin GPSmap 60CSx) and coordinated on the topographic map of Google Map (Map data 2020 Google). Map of geologic radon potential obtained from the Ministry of Industry, Department Mineral Resources (DMR) of Thailand (https://library.dmr.go.th/Document/DMR_Technical_Reports/2548/36805.pdf). Map of Thailand obtained from the Nations Online Project (https://www.nationsonline.org/oneworld/map/thailand-region-map.htm). Map of (**b**) adapted from map obtained from web site jAlbum.net (https://patricklepetit.jalbum.net/CHIANGMAI/MAPROOM/1476-072X-8-36-1-l.jpg).

**Figure 2:**
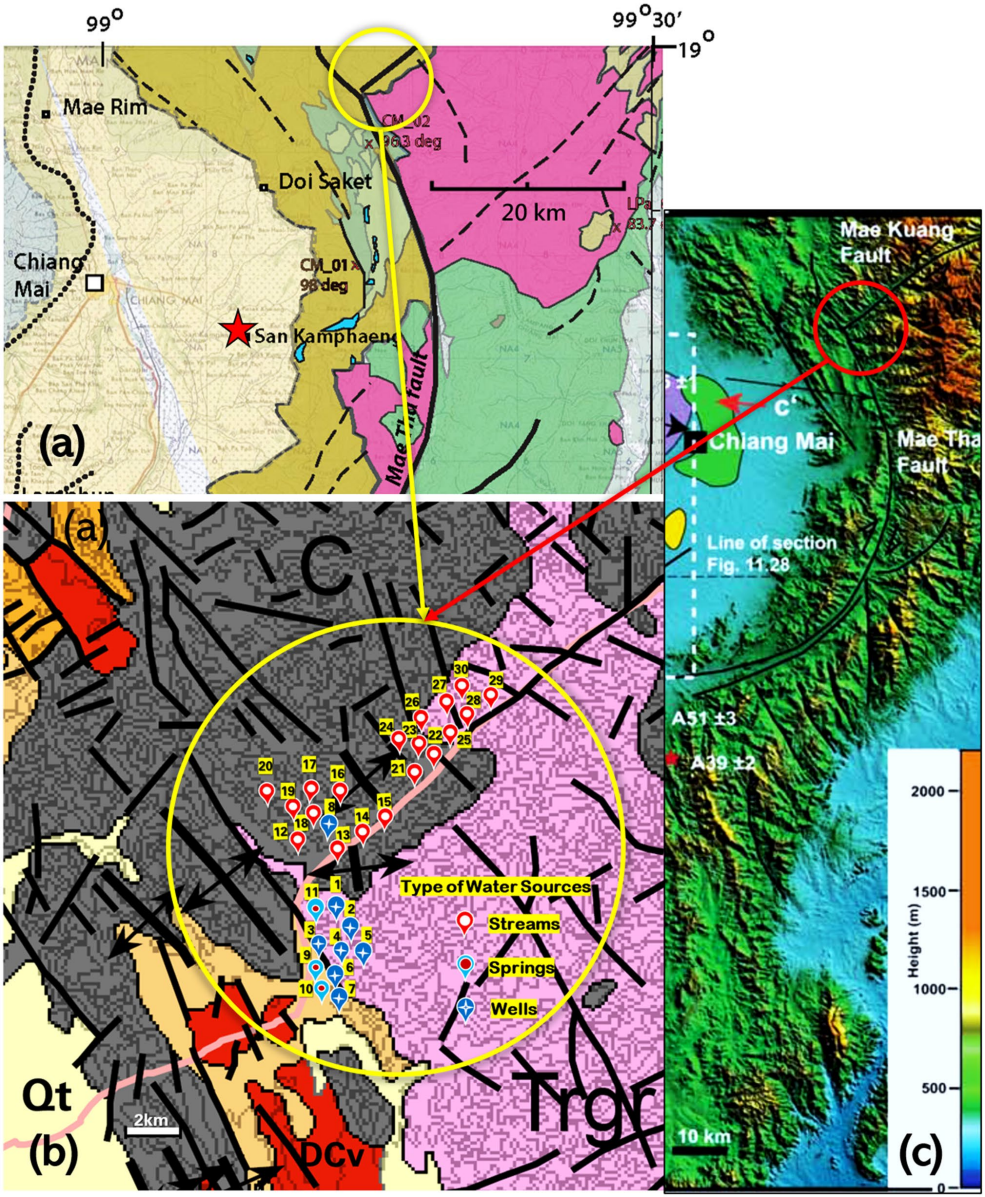
Sampling site locations (yellow circles) marked on geologic map29 (**a**), and tectonic lineaments map (**b**) of the Pa Miang Chiang Mai basin. Units include *Trgr* Triassic granite (represented by pink colour), *C* carboniferous conglomerate sandstone and shale (represented by gray colour), *Qt* quaternary terrace sediments (represented by yellow colour), DCv Basalt and tuff (represented by red colour). Black lines are mapped faults, some of which are based on topographic lineaments in the granitic rocks. (**c**) The elevation of the study locations (red circle) showed on topographic slope map.28 (**b**) Obtained from the Ministry of Industry, Department Mineral Resources of Thailand (https://www.dmr.go.th/ewtadmin/ewt/dmr_web/download/pdf/North/Chiangmai.pdf). See large illustrations of (**a**,**c**) in Supplementary Figs. S21 and S3, respectively.

Information concerning the study areas, sites and sampling locations marked on a geology map of the Doi Saket district are shown in Figs. 1 and 2.

## Results and discussion

All measurements from the field surveys at three sub-districts in Pa Miang (Ban Pong Kum [BPK], Ban Mae Wan [BMW], and Ban Pang Fan [BPF]) including the source of water supply to the residences, (wells, springs, streams), depth of groundwater wells, radon concentration in water, indoor radon-in-air in the living rooms and in the bedrooms, occupational time, building material, and number of windows is presented in Supplementary Table S1. The results of all calculation are presented in Supplementary Tables S2–S4.

**Figure 3:**
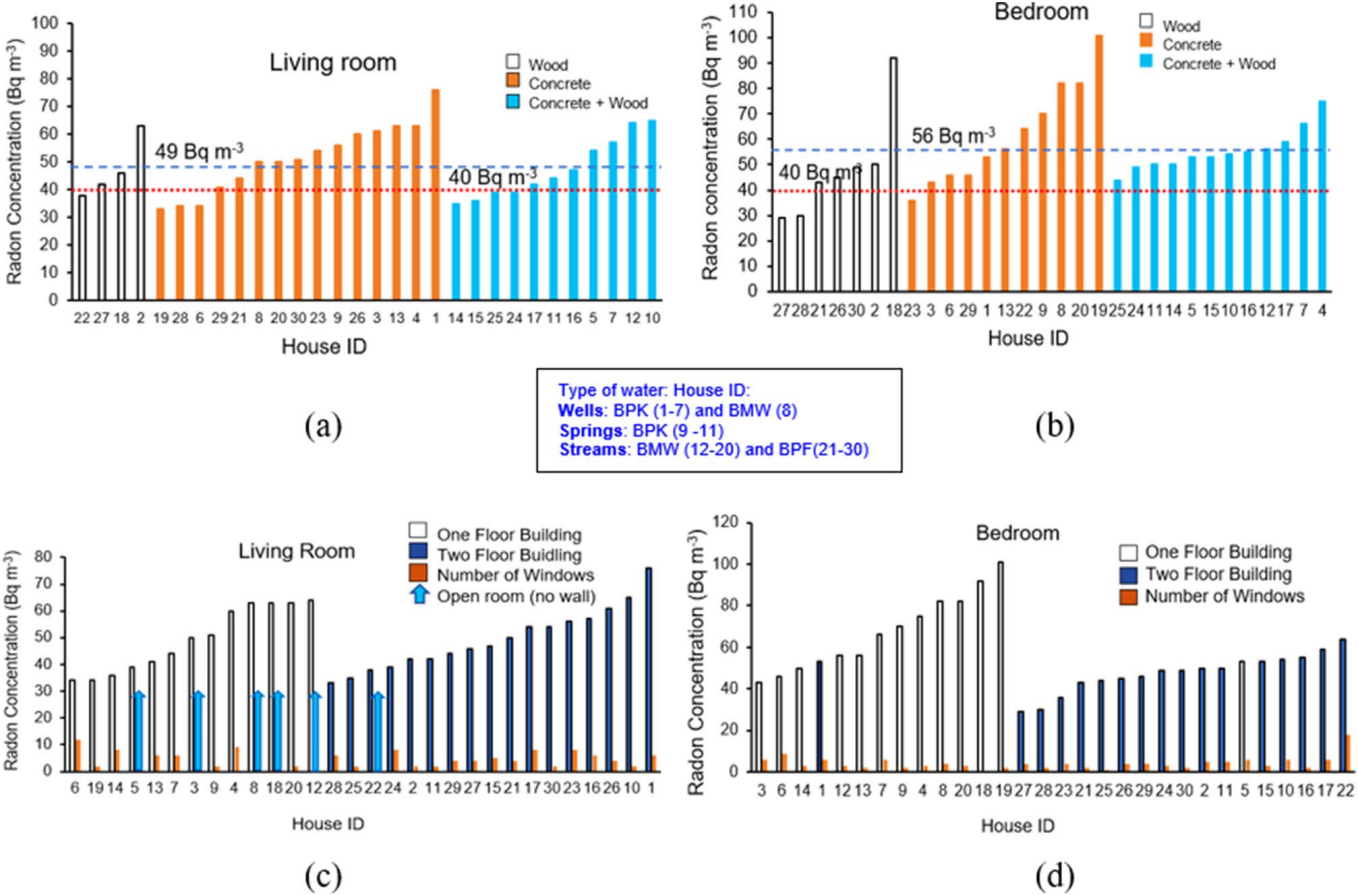
(**a**) Radon concentrations in living rooms using different building materials. The radon concentration ranged from 33 to 76 Bq m^−3^ with a mean of 49 Bq m^−3^ (represented by the blue long dash line). The red short dashed line represents the worldwide-average of 40 Bq m^−3^. (**b**) Radon concentrations in bedrooms using different building materials. The radon concentrations ranged from 29 to 101 Bq m^−3^ with a mean of 56 Bq m^−3^ (represented by the blue long dash line). The red short dashed line represents the worldwide-average of 40 Bq m^−3^. (**c**) A plot of radon concentrations in living rooms and (**d**) in bedrooms that located on the ground floor and second floor, and the number of the windows in the room.

The results of radon concentrations measured in the bedrooms and the living rooms in 30 houses ranged from 29 to 101 Bq m^−3^ and 33 to 76 Bq m^−3^, respectively. The average indoor radon concentration was 53 ± 15 Bq m^−3^ which is somewhat higher than the worldwide-average of 40 Bq m^−3^. More than 80% of the houses had higher concentrations than this worldwide-average value (Fig. 3a,b or see Supplementary Tables S2–S4). When considering a geologic source of radon at the study sites, this district is located in a known high radon potential area^27^ which is located in a basin of granitic rock that is rich in uranium and associated decay products. Moreover, the study areas are located in an active fault zone called the Mae Tha fault^28^ as shown in Fig. 2a,c, and Supplementary Figs. S21 and S3. The Mae Tha fault is a strike-slip fault, which is approximately 140 km-long, roughly NW-trending to the east of the Chiang Mai basin and the quartzose sandstones of the Mae Tha formation are extensively fractured. There are different types of rock units in the study areas including Triassic granites (Trgr); Quaternary terrace sediments (Qt); Carboniferous conglomerate sandstones and shales (C); and basalts and tuffs (DCv), that influences the radon in the study areas. Cho^30^ reported that the granitic gneiss such as Trgr, has the highest frequency ratios for high radon levels while sedimentary rocks, volcanic rocks, anorthosite, and some metasedimentary rocks (such as Qt, C and DCv), have low frequency ratios and low radon levels. In addition, areas of steep slopes and high elevation, like Pa Miang (Fig. 2c), which has high permeability due to faults and fractured rocks (Fig. 2b), allow radon to easily flow through fractures up to the surface and result in outdoor radon concentrations (and averages) of 39–57 (48 ± 5) Bq m^−3^, 36–70 (54 ± 12) Bq m^−3^ and 38–72 (54 ± 13) Bq m^−3^ were found in BPK, BMW and BPF, respectively. The outdoor radon can enter indoor environments through the open living areas, foundation of buildings, basements, and cracks. Thus, the radon concentrations measured from the open-air living areas of some houses are in the range of outdoor radon values. Due to Pa Miang’s geology and topography of high terrain (elevation range of 440–1760 m above sea level), outdoor values in the measurement areas were found to be much higher than the world average range of 5–15 Bq m^−3^^14^, and slightly higher than values found in the San Pha Tong district, which ranged from 12 ± 3 to 67 ± 10, with an average of 41 ± 2 Bq m^−3^^26^, approximately 60 km west of our study sites. San Pha Tong (red star symbol in Fig. 2a) has an elevation of about 293 m with gentlier slopes than at Pa Miang (Fig. 2c). Its geology is younger age with clay, silt, sand, and gravel, resulting in less radon contribution into the air than from the high permeable soils composed of faults and fractured rocks^30^ like Pa Miang.

When comparing the indoor radon activity concentrations found in this study with the other values measured in high background areas reported in Asia region based on similar methods and number of data collections as our study (Table 2), the highest value found in this study was much lower than those found in the high background areas in China, India and Indonesia but were in the range values found in another district of Chiang Mai^26^.

**Table 2:** Indoor radon reported in some Asia countries.

Country	Studied area	No. of dwelling	Range of radon concentration (average) Bq m^−3^	Type of building/material	Remarks	References
China	Yangxi and Yangdong Districts High background radiation areas : deposit of monazite sand supplied from nearby mountains	59	27–476 (124 ± 78)	Not avialable		Kudo et al.^44^
India	High natural background radiation area on the southeastern coast of Odisha: deposit of monazite sand	62	2–333 (91)	Cement, brick, and mud houses	Detectors were hung 0.2–2.0 m from the wall and 0.3–1.6 m from the ceiling in the bedroom or living room and some in the dining room or storeroom	Ramola et al*.*^45^
Indonesia	Takandeang village, Mamuju: hight background radiation	45	42–490 (221 ± 30)	Wood, unfired bricks, and mixed type	on the ceiling at a height of 200 cm from the floor and 100 cm from the wall of living room	Saputra et al*. *^46^
Thailand	Chiang Mai (Muang, Hang Dong, Saraphi and San Pha Tong)	55	35–209 (57 ± 2)	**–**	height of 1.0–2.0 m and 0.2 m from the wall in the bedroom	Autsavapro-mporn et al.^26^
Thailand	Chiang Mai (Pa Miang Doi Saket)	30	30–101 (53 ± 15)	Wooden, concrete and mixed types	Away from doors, windows or electric devices, walls and at about 2 m height above the floor	This Study

With respect to the type of room and ventilation (see Supplementary Table S1), as well as the sources of water used in those houses, it was found that average indoor radon concentration in the houses was somewhat different in each room type. The data revealed no significant linear correlation between the radon concentrations in the living room and the bedroom (r^2^ = 0.06, p > 0.05) (Supplementary Fig. S1). Nevertheless, considering the type of room and its radon concentration, the concrete-built room was potentially a contributor to higher radon more than in wood and concrete-wood built materials (Fig. 3a,b). Besides, most homes in the study area use air conditioning (data from interviews), so windows would normally be closed while in the house and would prevent dust entering the houses. As a result, the number of the windows did not influence the radon concentration in the rooms (Fig. 3c,d). It is likely that occasional high radon concentrations (e.g., House ID BPK 9 and BMW19) were the result of inefficient ventilation and use of concrete-built rooms as we observed in a previous study^31^. In addition to building materials, reduced window opening behavior that correlated with increased radon concentration, the geology of the area, topographic information and the design of the house (one or two stories) can influence radon levels. The type of houses in the study areas is a typical Thai style house. Every house has a living area (or living room) on the ground floor, some spaces are partial or fully open-air (no enclosing wall), and some have dirt (earthy) floors. Radon that diffuses directly from the ground is the primary source of radon in these living areas such as in the case of house ID BMW-18 which is made of wood and has a dirt floor. Radon levels were found to be remarkably high in the living area of this house (Fig. 3c). Moreover, radon from a dirt floor or ground floor can more easily leak through the wooden floor into the bedroom than would be the case in a multi-story concrete-built house (Fig. 3d). Therefore, the design of the house, the ventilation conditions and geology/topography can enhance or reduce the indoor radon concentrations. In some cases, open-air living areas have radon levels higher than the average indoor values.

**Figure 4:**
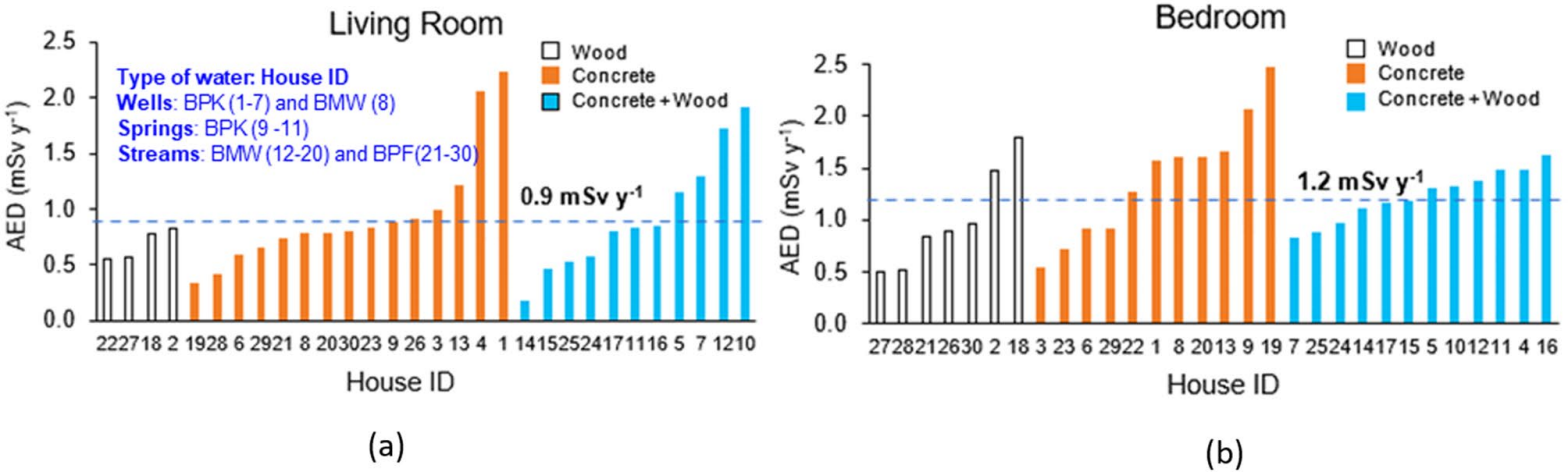
(**a**) AIED in living rooms using different building materials. The AIED for residents in the living room ranged between 0.2 and 2.2 mSv year^−1^ with a mean of 0.9 mSv year^−1^ (represented by the blue long dashed line). (**b**) AIED in bedrooms using different building materials. The AIED for residents in the bedroom ranged between 0.5 and 2.5 mSv year^−1^ with a mean of 1.2 mSv year^−1^ (represented by the blue long dashed line).

To estimate the AED due to inhalation of radon in the air, in addition to the radon concentration, the occupancy time (*T*) in the building is an important parameter in the calculation of inhalation AED Eq. (2). In this study, from interviews with the owners of the houses, we found that some homes have elderly people staying at home all the time. So for AED calculations, we chose the longest time people spent in their homes because they are most affected by indoor radon, resulting in the *T* value was found to range from 11 to 24 h day^−1^ with an average of 16 h day^−1^ (Supplementary Table S1). These times were used to calculate the AED. The AIED of the residents stayed in the living rooms ranged between 0.2–2.2 mSv year^−1^ with a mean of 0.9 mSv year^−1^ and 0.5–2.5 mSv year^−1^ with a mean of 1.2 mSv year^−1^ in bedrooms (Fig. 4a,b). The estimated total AIED received from radon in living rooms and bedrooms ranged from 0.9 to 3.8 mSv year^−1^ with the mean of 2.1 mSv year^−1^ (Table 3). These doses were much less than the recommended maximum indoor radon doses of 14 mSv year^−1^ for the public^15^. This was expected as the indoor radon concentrations found in this study were less than the upper reference level of 300 Bq m^−3^ used by ICRP − 126^14^.

**Table 3:** Summary of AIED of radon concentration in living room, bedroom, and the percent contribution from water from different sources to indoor radon.

Source of water	Number of samples	AIED (mSv year^−1^)					AIED of Rn degassed from water into air (mSv year^−1^)		Percent contribution (%)	
		Living room		Bedroom		Total (Min–Max)				
		Min–Max	Average	Min–Max	Average		Min–Max	Average	Min–Max	Average
Wells	8	0.6–2.2	1.2	0.5–1.6	1.2	1.5–3.8	0.04–0.5	0.2	1.6–13.0	10
Springs	3	0.8–1.9	1.2	1.3–2.1	1.6	2.3–3.2	0.1–0.3	0.2	2.2–13.0	6.0
Streams	19	0.2–1.7	0.7	0.5–2.5	1.2	0.9–3.1	0.4 × 10^–3^–4.2 × 10^–3^	1.6 × 10^–3^	0.03–0.2	0.1
All water sources	30	0.2–2.2	0.9	0.5–2.5	1.2	0.9–3.8	0.4 × 10^–3^–0.5	0.1	0.03–13.0	3.3

**Figure 5:**
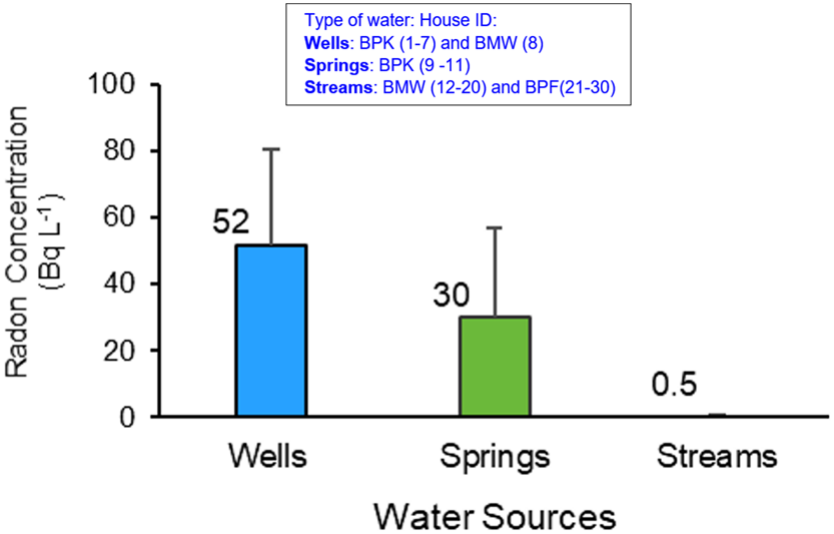
Average radon concentrations and standard deviations from various sources of water analyzed in this study.

The data of the source of water supply collected from 30 sites including 8 private wells, 3 springs (waterfalls) and 19 streams waters are showed in the Supplementary Table S1. Average radon concentrations from various sources of water analyzed are shown in Fig. 5. The result of radon concentration values in eight private wells ranged from 11 to 84 Bq L^−1^, with a mean value of 52 Bq L^−1^. Well No.1 (BPK1) located in the BPK site had the highest radon level. We observed that similar radon values were found at the same well depth and tended to decrease with the depth of the well. The reason for the similarity of the value at same depth might be due to similar pathways shared by these waters.

For the 19 water supplies, that use water from streams, the radon concentration values were lower and ranged from 0.1 ± 0.02 to 1.1 ± 0.5 Bq L^−1^, with a mean value of 0.5 ± 0.3 Bq L^−1^. The 3 water supplies originating from the spring-fed waterfalls ranged from 13.3 ± 1.8 to 60.9 ± 14.2 Bq L^−1^ with an average of 30.1 ± 6.1 Bq L^−1^. The radon concentration in water supplies using water resources from streams in BMW were slightly less than those from BPF. Although the water from these streams was treated using the same method but they came from different mountains. We observed that the average radon activities in the water from the wells and springs collected from the BPK site were much higher than those from the streams collected from BMW and BPF (Fig. 5). This is a characteristic of well water and spring water (waterfall) in that they normally have radon activities that are higher than surface waters, including streams, since they are in contact with geological formations rich in uranium, such as granite rocks, present in our study area. In addition, sometimes in a closed or nearly closed system, like a shallow well, radon can accumulate and reach activity concentrations of several kBq L^−1^^32^. In addition, the water supplies used at BPK delivered the water directly to houses by pumping from the well or flowing through bamboo trunks, without being processed through any water treatment system. This results in a short distance and time between the water source and the end user. Therefore, any decrease in radon due to decay and degassing were minimal. This is in contrast to the tap water collected from the other two village sites where the water from the streams was pumped to collect in a tower and subsequently treated before distributing to homes.

Comparison of radon activity concentrations among the different water types, the radon activity levels found in this study were within the commonly reported ranges found elsewhere (Table 4). In addition, this study showed that the groundwater sources, including the springs and wells, some of which are derived from granitic bed rocks, have higher radon activity levels than the surface waters (rivers and streams). When comparing the radon concentrations in these waters with the international recommended values, all samples were found to be lower than the 100 Bq L^−1^ threshold recommended by the European Union Commission for drinking water. However, all well and spring water samples collected in BPK had radon concentrations higher than the U.S. EPA MCL (11.1 Bq L^−1^) of radon in drinking water but less than the U.S. EPA AMCL of 148 Bq L^−1^ limit for radon in water. Thus, it would not be necessary to introduce the MMM program to address radon risks in indoor air, as suggested by the U.S.EPA.

**Table 4:** Radon activity concentrations from different water sources.

Water type	Radon concentration (Bq L^−1^)	Country, region	Geology/source	References
Drinking water	1.46–644	Austria	Granite bedrock	Wallner and Steininger^47^
	1.9–112.77	Portugal	Tap	Lopes et al.^48^
	0.19–71.1	UK	Tap	Henshaw et al.^49^
Tap water	1–2	United Kingdom		Mustafa et al.^50^
	1.2–4.5	Romania	A post-tectonic depression	Nita et al.^51^
	0.18–1.13	Doi Saket, Chiang Mai, Thailand	Igneous and sedimentary rocks/ stream originated from mountain	This study
Groundwater and Well water	3800	Finland	Soil (no detail)	Salonen^52^
	1220	Germany, east Bavaria	Granite, gneiss	Trautmannsheimer et al.^53^
	17–3856	Portugal, Nisa	Granites, sediments	Pereira et al.^54^
	4–63,560	Sweden, Stockholm County	Various, crystalline bedrock	$Skeppstr\ddot{O} m$ and Olofsson^55^
	77,000	Finland	Mainly granitic bedrock	Salonen^52^
	0.1 to 483.0	Namom district, Thailand	Triassic granite, Carboniferous shale, and Quaternary sediment	Pisapak and Bhongsuwan^56^
	5.6–35.2	Romania	A post-tectonic depression	Nita et al.^51^
	11.38–84.20	Doi Saket, Chiang Mai, Thailand	Igneous and sedimentary rocks	This study
Spring and non-bottled mineral waters	1.4–105	Spain, South Catalonia	Volcanic (granite) and sedimentary rocks (e.g. limestone, sandstone)	Fonollosa et al.^57^
	2.11–120	Hungary, Balaton Highland, South Transdanubia and the South Great Plain	Sedimentary rocks	Somlai et al.^58^
	1.4–43.7	Lithuania	Crystalline and sediment rocks	Ladygiene et al.^59^
	1029	Spain, Galicia	Granitic and slate rocks	Llerena et al.^60^
	10.2–68.9	Romania	A post-tectonic depression	Nita et al.^51^
	13.29–60.91	Doi Saket, Chiang Mai, Thailand	Springs (waterfall) originated from mountain	This study
Hot spring	53.4–292.5	China	Granite bedrocks	Song^61^
	0.79–76.53	Mae Hong Son, Chiang Mai, Chiang Rai and Lampang, Thailand	Igneous and sedimentary rocks	Wanapongse et al.^62^
	2–154	Suan Phueng district, Ratchaburi province, Thailand	Igneous and sedimentary rocks	Sola et al.^63^

**Figure 6:**
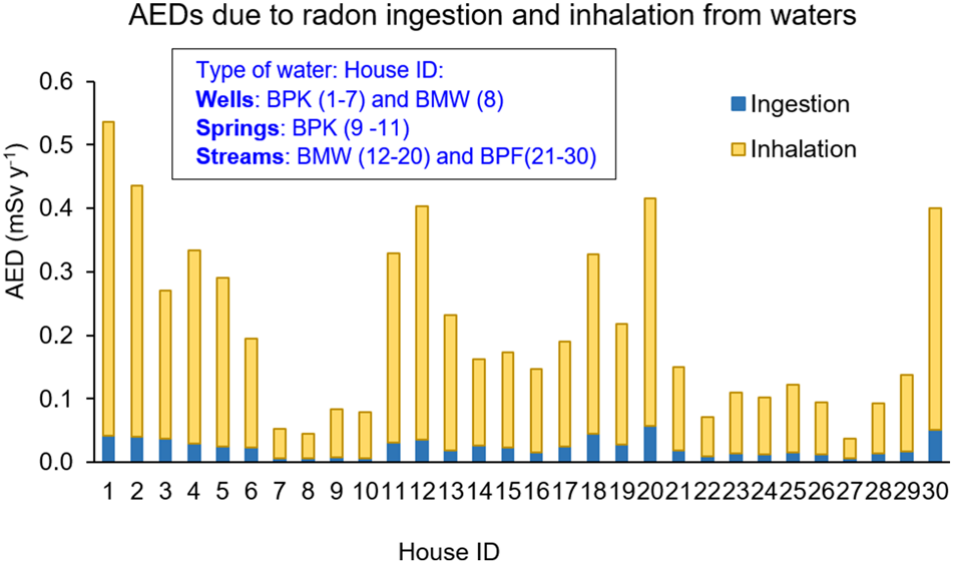
AEDs due to radon ingestion and inhalation from waterborne radon. Note that the units for house ID 12–30 is 10 ^−2^ mSv year^−1^. The house ID 1–8 are collected from wells, ID 9–11 collected from springs and ID 12–30 collected from streams.

In order to evaluate the ED caused by drinking waterborne radon, we made certain assumptions that, for well water, any radon released from its subsurface geology to the well waters is continuous and constant over time and that an equilibrium radon concentration was established. We also assumed that the well water was directly consumed, so the radon would have no opportunity to be lost from the water by heating or boiling. For the total annual water intake, we used the maximum value (2 L day^−1^ or 730 L year^−1^) taken from the study of Sawangjang et at.^33^ to calculate the ingestion ED according to Eq. (4). The calculated results for AED due to ingestion of water from wells, springs and treated stream sources ranged from 0.01 to 0.04 mSv year^−1^, 0.7 × 10^–2^ to 3.1 × 10^–2^ mSv year^−1^, and 0.1 × 10^–3^ to 0.6 × 10^–3^ mSv year^−1^, respectively (see Supplementary Tables S2–S4). The AED due to ingestion (see Fig. 6) from all water sources was found to be lower than the IDC of 0.1 mSv year^−1^ suggested by the WHO for drinking water consumption for the public.

Considering the fraction of the initial radon concentration in water (Bq L^−1^) that contributed to the indoor-airborne radon concentration (Bq m^−3^), our results found that the contribution from radon degassed from water into the house air ranged from 1.1 to 8.4 Bq m^−3^, 1.3–6.1 Bq m^−3^, and 0.01 to 0.11 Bq m^−3^ for water from the wells, springs, and streams, respectively (Supplementary Tables S2–S4). Using Eq. (3). the results show that the AED associated with inhalation of degassed radon from water to indoor radon ranged from 0.04 to 0.5 mSv year^−1^, 0.1 to 0.3 mSv year^−1^ and 0.3 × 10^–3^ to 4.0 × 10^–3^ mSv year^−1^ for wells, springs, and treated streams, respectively (Table 3) The highest total ED (ingestion plus inhalation of radon) of 0.5 mSv year^−1^, was via consumption of well water. These results show that radon in water can add a small yet significant amount of radon to indoor air, and the associated additional radon inhalation doses are much higher than the radon ingestion dose. In other words, the health risks resulting from inhalation of radon released from water to air were considered to be significantly higher than the risks associated from the consumption (ingestion) of radon in drinking water (Fig. 6).

We also investigated the proportion of inhalation ED from radon released from water contributed to the residents living rooms and bedrooms. The results found that the fraction of AED from inhalation of radon degassed from water that originated from wells, springs, and treated streams were about 10%, 6%, and 0.1% of the total AIEDs, respectively, (Table 3). Even though our study found that all tested water was safe to be consumed (at 2 L day^−1^), with respect to the radon content in the water and associated health risk, it is suggested that waters from wells and springs should be either passed through an appropriate filtration system or be stored to let radon escape or decay before use.

## Conclusions

Radon is known as the leading cause of lung cancer mortality and incidence after smoking in Chiang Mai, Thailand. To find a source of carcinogens, which is one of the important measures for helping to prevent the cancer risk, specific measurement sites at Pa Miang, Doi Saket were selected. These study sites have several strong combination factors influencing indoor radon concentration, including different topographical and geologic features associated with active faults, and mountain terraces. These factors make outdoor radon concentrations in the study area higher that the global average^14^ and are the main sources of indoor radon for the studied wooden houses. Our results indicated that besides building materials, the design of the house, ventilation conditions and lifestyle choices are all factors influencing indoor radon concentrations and associated risks. The actual time people spend at home is also an important factor, as the calculated AEDs were found to be high in homes with elderly people.

We found from studies of internal dose obtained from ingestion of drinking water from different sources and radon contributed by degassing from well water contributed about 10% of the total AIED. Although radon levels (29–101 Bq m^−3^) and total indoor annual effective doses (0.9–3.8 mSv year^−1^) received from all sources in these areas showed no significant health risk due to exposure of radon, the spatial analysis studies should be useful as a starting point in understanding the behaviour of indoor radon and guiding strategies to prevent the risk of lung cancer in Chiang Mai. However, in order to achieve an improved statistical analysis, further investigations should be carried out with a larger number of samples.

## Methods

### Study design

Our study was conducted in the areas of Chiang Mai province, Thailand. The study consisted of field measurements and data collection by interviews. All experimental protocols were approved by the committee of the National Research Council of Thailand (NRCT) and carried out in accordance with the relevant guidelines and regulations of that agency. All participants, including those interviewed, were informed of the purpose of the study, its potential benefits and risks or inconveniences that could result from the study protocol. A structured questionnaire was administered in the Thai language to 30 residents who gave their consent to be part of the study. Informed consent was obtained from all participants after the nature of the study was explained and prior to completing the questionnaire, and at the time of sample collection.

### Study area

#### Geological background

Thailand is located in Southeast Asia bounded to the west by Myanmar, to the north by Myanmar and Laos, to the east by Laos and Cambodia, and to the south by Malaysia. Physiographically, the country can be divided into four regions; the mountainous highland in the north and northwest, the khorat plateau in the northeast, the central plain and the southern peninsular, which is between the Andaman Sea and the Gulf of Thailand^34^.

The study area was located in Chiang Mai province within the basins of northern Thailand. These basins are mainly covered with Quaternary alluvial and terrace deposits and lay in an active fault zone called the Mae Tha fault (Fig. 2c), approximately 140 km-long, roughly NW-trending to the east of the basin. The sedimentary, igneous and metamorphic rocks surrounding the basins range from Silurian–Devonian to Permian Groups. Doi Saket (Fig. 1b), a district of Chiang Mai (Northern Thailand) was selected as the study area based on its geology, that includes a combination of rock units; Trgr, C, Q, and DCv; and is associated with many fault lines and fractures^27–29^, resulting in an area of high radon potential (Figs. 1a, 2). This is based on interpretation of airborne radiometric surveys performed by the Department of Mineral Resources of Thailand during 1985–1987^27^. Moreover, this district was reported as one of the highest incidences of lung cancer cases among the 25 districts of Chiang Mai^26^.

The people living in Doi Saket area still use water supplied from the granitic highland mountains for their daily use and ingestion. Doi Saket has 14 sub-districts, from which we selected Pa Miang. However, Pa Miang is comprised of six villages that cover approximately 160 km^2^ and most of the terrain is mountains, highlands, and valleys at approximately 440–1760 m above mean sea level with 1463 households and approximately 3500 inhabitants. Thus, three villages in Pa Miang (Ban Pong Kum [BPK], Ban Mae Wan [BMW], and Ban Pang Fan [BPF]) were selected as subsites for measurement of the radon concentrations in water supplies and indoor air in the houses. The geology of BPK and BPF is mainly igneous rocks with Trgr type (biotite, tourmaline, and muscovite-biotite granites) while the geology of BMW is mostly sedimentary and metamorphic rocks (shale, chert and limestone) with C type lines on the top of Trgr. BPK is 45 km from Chiang Mai city and has 486 houses, BMW which is 10 km from BPK and 20 km from BPF, has 242 houses and BPF has 245 houses.

#### Building materials

The type of house in the study areas is a typical Thai style house with one or two-stories. The size of the rooms in each house are not large, about 12–30 m^2^. The windows are made of glass or wood. Almost all two-story houses have walls on the first floor made of concrete, while the second floor with bedrooms, have floors made of wood and the walls are made of wood or a combination of concrete and wood. Every two-story house has a living area (or living room) on the ground floor, and some spaces are partial or fully open-air (no enclosing wall). Most one-story houses have walls made of concrete or a combination of concrete and wood. A one-story house with a wood floor raised above the ground often uses the open space under the house as a living room.

To identify the indoor radon sources of the residents and its associated radiological risk, the indoor radon concentrations and those in water were measured in thirty volunteer houses of the selected areas, as discussed below.

### Indoor Radon measurements

The indoor ED of the residents from all radon sources in the house was determined using solid state alpha track-etch film detector (CR-39) manufactural by Nagase Landauer, Ltd. (Ibaraki, Japan), which placed in the case called “RADUET”, manufactural by Radosys, Ltd., Hungary^35^. Since the size of the rooms are not large (12–30 m^2^), one CR-39 detector was installed in each room. A total of 60 CR-39 detectors were installed in the same houses where the water was sampled and were placed in the bedroom and living rooms away from doors, windows, or electrical devices, at about 2 m height above the floor. Detailed information concerning the house description, numbers of windows, and the occupancy time spent in each room (*T*) where the detectors were installed were collected by interviewing the house owners. After leaving the detectors at the houses for 120 days, all the exposed detectors were collected and chemically etched for 24 h in 6.25 M sodium hydroxide solution at 60 °C^36–38^, and then washed and dried before counting the track density (tracks mm^−2^) by optical microscopy to evaluate the radon concentration. The uncertainty (less than 3% for k = 2) of the track radon activities was conducted in the inhouse radon calibration chamber, which was verified through the international intercomparison project^39,40^.

### Types of water sources

The water supplies in the study area comes from three sources: well, springs, and streams waters as discussed below.

#### Well waters

People living in BPK and BMW used private shallow well waters to supply their household water. The depth of the wells ranged from 4 to 14 m below the surface. Each well was lined with cement and closed with a concrete lid. Electric pumps were used to retrieve the groundwater when needed. A total of eight well water samples were collected; seven from the BPK site (BPK1-7) and one well from the BMW site (BMW8). No well waters were used in the BPF site.

#### Springs

The source of tap water supply for the BPK site is a waterfall arising from springs on the nearby mountain. Bamboo pipes were used to deliver water from the waterfall reservoirs on the mountain and distributed directly to the houses for consumption without any treatment or processing. A total of three water samples (BPK9-11) were collected from water taps that belong to the volunteer houses in the BPK site.

#### Stream waters

The sources of tap water supplies for households in BMW and BPF are water streams. These streams originate from the mountains nearby the villages. The water from these streams are pumped and collected in concrete storage towers that belong to the villages. The water is then processed through a water filtration system, before being distributed to the individual houses. A total of 19 water samples, consisting of nine from the BMW site (BMW12-20) and ten from the BPF site (BPF21-30), were collected from water taps belonging to the volunteer houses.

### Sample collection and analysis

A total of 30 water samples were collected from the different volunteer houses distributed within three villages in the study area (Fig. 1c). To avoid a temperature effect on the ^222^Rn activity concentrations, the water samples were all collected in February 2018, when the water temperature ranged from 22 to 29 °C. The samples were taken at the points of consumption, as suggested by the WHO^21^, and were directly collected into the 250-mL bottles used by the RAD-H2O accessory of the RAD-7, an electronic radon detector^41^. Three bottles of water sample were collected for each sample point and care was taken to exclude any air bubbles. Since the RAD-7 system is portable, the measurements of radon-in-water were performed in the field. During a 5-min aeration of a 250-mL water sample in a closed loop, more than 95% of the available radon is removed from the water. Triplicate measurements indicated that the precision of this method was approximately 10%. After each analysis, the system was purged with dry air for approximately 30 min before the next measurement was conducted. The relative humidity was maintained at less than 10% for each analysis. Due to the short ^222^Rn half-life (3.82 days), decay correction must be considered from when the measurement starts and collection time or any other delay in the measurement after sampling. The measured radon gas activity was corrected back to the collection time based on the standard equation of radioactive decay as shown in Eq. (1);

1 $$A = {A}_{0} {e}^{-(\lambda t)}$$

where *A*_*0*_ is the initial activity (BqL^−1^), *A* is the measured activity (BqL^−1^), *λ* is the radon decay constant (per minute) and *t* is the period between the collection and the testing (min).

### Health risk assessment: AEDs

Radon can enter the human body by ingestion through the gastrointestinal tract and by inhalation through the respiratory tract. Both mechanisms constitute potential health hazards. Hence, the annual health risk or the annual indoor ED (AIED) (in the units of mSv year^−1^) for the general public will be the sum of EDs from all the sources by ingestion (E_ing_) of drinking waterborne radon, and inhalation (E_inh_) of radon in the house (indoors). We used several parameters currently recommended by the WHO and ICRP to calculate the EDs as follows.

#### Inhalation risk

The annual indoor ED (AIED) of the residents due to inhalation of radon from all sources in air was calculated using Eq. (2).

2 $${E}_{inh} = {C}_{Rn} \times T \times {DCF}_{inh}$$

where *C*_*Rn*_ is the radon activity concentration in the room (Bqm^−3^), *T* is the average indoor occupancy time, and *DCF*_*inh*_ is the dose conversion factor or ED coefficient equal to 6.7 × 10^–6^ mSv/(Bq hm^−3^), if using an equilibrium factor between radon and its progeny equal to 0.4^42^. Note that since values for the equilibrium factor (F) specifically for the conditions in the measurement areas were not available, the default value 0.4 recommended by ICRP − 137 was used in the AIED calculation.

The AED due to inhalation of radon (E_inh_) degassing from water was estimated using Eq. (3);

3 $${E}_{inh} = {C}_{Rn} \times {R}_{aw} \times T \times {DCF}_{inh}$$

where *R*_*aw*_ is the transfer coefficient of radon from water to the air. We used a value of 10^–4^ or 0.1 Bq m^−3^/(Bq L^−1^)^43^.

The AIED is a factor that not only consider the indoor radon concentration but also the time people spend in the house. Thus we obtained the house occupancy time (*T*) in Eq. (3), from resident interviews. This was used in the calculation for both AIED of radon degassed from water and the AIED of the indoor air. We considered this more accurate than using an average of 7,000 h year^−1^ or 19.18 h d^−1^, recommended by the UNSCEAR^2^.

#### *Ingestion risk (E*_*ing*_*)*

The AED due to ingestion (E_ing_) of radon in water was estimated according to parameters introduced by the WHO and ICRP report^6^, using Eq. (4);

4 $${E}_{ing} = {C}_{Rn} \times {C}_{w} \times {DCF}_{ing}$$

where *C*_*Rn*_ is the radon activity concentration in water (Bq L^−1^), *C*_*w*_ is the yearly water consumption rate (L year^−1^), and was taken to be 2 L day^−1^, and *DCF*_*ing*_ is the dose conversion factor or ED coefficient per intake of ingested radon, equal to 6.9 × 10^–7^ mSvBq^−1^^15^.

## Supplementary information

Supplementary Information

## Data Availability

All data generated and analysed during this study are included in this published article and its Supplementary Information files.
