# Supplementary material for: Identifying indoor radon sources in Pa Miang, Chiang Mai, Thailand
Source: Sci Rep. 2020 Oct 20;10:17723. doi: 10.1038/s41598-020-74721-6 (PMC7576592; doi:10.1038/s41598-020-74721-6)
Supplement: Supplementary file 1 — Supplementary Information [file 41598_2020_74721_MOESM1_ESM.pdf]

SUPPLEMENTAL IMFORMATION

Identifying Indoor Radon Sources in Pa Miang, Chiang Mai, Thailand

Tarika Thumvijit[1,2], Supitcha Chanyotha[3,4,*], Sompong Sriburee[1,2], Pongsiri Hongsriti[1], Monruedee Tapanya[1], Chutima Kranrod[3,4,5] and Shinji Tokonami[5]

[1]Department of Radiologic Technology, Faculty of Associated Medical Sciences, Chiang Mai University, Chiang Mai, 50200 Thailand

[2]Center of Radiation Research and Medical Imaging, Chiang Mai University, Chiang Mai, 50200 Thailand

[3]Department of Nuclear Engineering, Faculty of Engineering, Chulalongkorn University, Bangkok, 10330 Thailand

[4]Natural Radiation Survey and Analysis Research Unit, Chulalongkorn University, Bangkok, 10330 Thailand

[5]Department of Radiation Measurement and Physical Dosimetry, Institution of Radiation Emergency Medicine Hirosaki University, Hirosaki, Aomori, 036-8564 Japan

***Corresponding author**
Supitcha Chanyotha
E-mail: supitcha.c@chula.ac.th

Supplementary Materials:

Supplementary Table S1 presents all measurements from the field surveys including the source of water supply to the residences, (well, springs, streams), depth of groundwater wells, radon concentration in water, indoor radon-in-air in the living rooms and in the bedrooms, occupational time, building material, and number of windows.

Supplementary Tables S2-S4 present the radon concentration degassed from water derived from wells, springs and streams into indoor air, AED from drinking well water and total AIED received by occupants staying in the houses using water supplies from those type of waters.

Supplementary Figure S1 presents data points plotted against radon concentration in the living rooms and the bedrooms with linear regression.

Supplementary Figure S2. presents the geologic map of the Chiang Mai basin

Supplementary Figure S3 presents the Mae Tha fault and active faults and seismic activity zones in Thailand.

SUPPLEMENTARY TABLE S1. All measurements from the field surveys including the source of water supply to the residences, (well, springs, streams), depth of groundwater wells, radon concentration in water, indoor radon-in-air in the living rooms and in the bedrooms, occupational time, building material, and number of windows.

Source of water	House ID.	Well depth (m.)	*Rn conc. in water (Bq L^{-1})	Living room (LR) **Rn conc. (Bq.m^{-3})	LR T (hr.)	LR Room Type	LR Number of windows	Bedroom (BR) **Rn conc (Bq.m^{-3})	BR T (hr.)	BR Room Type	BR Number of windows	Number of floors
Private wells (n=8)	BPK1	14	84.2	76	12	C	6	53	12	C	6	2
	BPK2	14	80.9	42	8	W	2	50	12	W	5	2
	BPK3	13	73.3	50	8	C	open	43	5	C	6	1
	BPK4	10	56.8	60	14	C	9	75	8	C+W	3	1
	BPK5	10	49.5	39	12	C+W	open	53	10	C	6	1
	BPK6	10	46.8	34	7	C	12	46	8	C	9	1
	BPK7	4	11.4	44	12	C+W	6	66	5	C+W	6	1
	BMW8	4	12.0	63	5	C	open	82	8	C	4	1
	Average	10	51.9	51	7.3			58.2	8.5			
	Min-Max	4-14	11.4- 84.2	34-76	5-14			43-82	5-12			
Springs (n=3)	BPK9		16.1	51	7	C	2	70	12	C	2	1
	BPK10		13.3	65	12	C+W	2	54	10	C+W	6	2
	BPK11		60.9	42	8	C+W	2	50	12	C+W	5	2
	Average		30.1	52.7	9			58	11.3			
	Min-Max		13.3-60.9	42-65	7-12			50-70	10-12			
Streams (n=19)	BMW12		0.7	64	11	C+W	open	56	10	C+W	3	1
	BMW13		0.4	41	12	C	6	56	12	C	2	1
	BMW14		0.5	36	2	C+W	8	50	9	C+W	3	1
	BMW15		0.5	47	4	C+W	5	53	9	C+W	3	2
	BMW16		0.3	57	6	C+W	6	55	12	C+W	2	2
	BMW17		0.5	54	6	C+W	8	59	8	C+W	6	2
	BMW18		0.9	63	5	W	open	92	8	W	no window	1
	BMW19		0.6	34	4	C	2	101	10	C	2	1
	BMW20		1.1	63	5	C	2	82	8	C	3	1
	BPF21		0.4	50	6	C	4	43	8	W	2	2
	BPF22		0.2	38	6	W	open	64	8	C	18	2
	BPF23		0.3	56	6	C	8	36	8	C	4	2
	BPF24		0.3	39	6	C+W	8	49	8	C+W	3	2
	BPF25		0.3	35	6	C+W	2	44	8	C+W	1	2
	BPF26		0.2	61	6	C	4	45	8	W	4	2
	BPF27		0.1	46	5	W	4	29	7	W	4	2
	BPF28		0.3	33	5	C	6	30	7	W	2	2
	BPF29		0.4	44	6	C	4	46	8	C	4	2
	BPF30		1.0	54	6	C	2	49	8	W	2	2
	Average		0.5	49	6			55	8.6			
	Min-Max		0.1-1.1	33-64	2-12			29-101	7-12			

*Detecting by RAD- H2O, **Detecting by CR-39, BPK = Ban Pong Kum site, BMW = Ban Mae Wan site, BPF = Ban Pang Fan site, C = Concrete, W = Wood, T = Time spent in the room. Open = Open-air room without walls.

SUPPLEMENTARY TABLE S2. The radon concentration degassed from water derived from wells into indoor air, AED from drinking well water and total AIED received by occupants staying in the houses using water supplies from wells.

Source of water	House ID	*Degassed into air (Bq m^{-3})	AED from drinking water (mSv y^{-1})			AIED (mSv y^{-1})		
			E_{ing}	E_{inh}	Total	Living Room	Bed room	Total
Wells (n=8)	BPK1	8.4	0.04	0.5	0.5	2.2	1.6	3.8
	BPK2	8.1	0.04	0.4	0.4	0.8	1.5	2.3
	BPK3	7.3	0.03	0.2	0.3	1.0	0.5	1.5
	BPK4	5.7	0.03	0.3	0.3	2.1	1.5	3.5
	BPK5	5.0	0.02	0.3	0.3	1.1	1.3	2.4
	BPK6	4.7	0.02	0.2	0.2	0.6	0.9	1.5
	BPK7	1.1	0.01	4.7×10^{-2}	0.1	1.3	0.8	2.1
	BMW8	1.2	0.01	3.8×10^{-2}	4.8×10^{-2}	0.8	1.6	2.4
	Average	5.2	0.03	0.24	0.3	1.2	1.2	2.4
	Min-Max	1.1-8.4	0.01-0.04	0.04-0.5	0.04-0.5	0.6-2.2	0.5-1.6	1.5-3.8

* Value obtained from radon concentration multiplied by the transfer coefficient (10^{-4}), BPK = Ban Pong Kum site, BMW = Ban Mae Wan site.

SUPPLEMENTARY TABLE S3. The radon concentration degassed from water derived from springs into indoor air, AED for ingestion and total AIED received by occupants staying in the houses using water supplies from springs.

Source of water	House ID	*Degassed into air (Bq m^{-3})	AED from drinking water (mSv y^{-1})			AIED (mSv y^{-1})		
			E_{ing} ($\times 10^{-2}$)	E_{inh}	Total	Living Room	Bed room	Total
Springs (n=3)	BPK9	1.6	0.8	0.1	0.1	0.9	2.1	3.0
	BPK10	1.3	0.7	0.1	0.1	1.9	1.3	3.2
	BPK11	6.1	3.1	0.3	0.3	0.8	1.5	2.3
	Average	3.0	1.5	0.15	0.2	1.2	1.6	2.8
	Min-Max	1.3-6.1	0.7-3.1	0.1-0.3	0.1-0.3	0.8-1.9	1.3-2.1	2.3-3.2

* Value obtained from radon concentration multiplied by the transfer coefficient (10^{-4}), BPK = Ban Pong Kum site.

SUPPLEMENTARY TABLE S4. The radon concentration degassed from water derived from streams into indoor air, AED from drinking tap water and total AIED received by occupants staying in the houses using water supplies from streams.

Source of water	House ID	Degassed into air (Bq m^{-3})	AED from drinking water (mSv y^{-1})			AIED (mSv y^{-1})		
			Eing ($x10^{-3}$)	Einh ($x10^{-3}$)	Total ($x10^{-3}$)	Living Room	Bed room	Total
Streams (n=19)	BMW12	0.07	0.4	3.7	4.0	1.7	1.4	3.1
	BMW13	0.04	0.2	2.1	2.3	1.2	1.6	2.8
	BMW14	0.05	0.3	1.4	1.6	0.2	1.1	1.3
	BMW15	0.05	0.2	1.5	1.7	0.5	1.2	1.6
	BMW16	0.03	0.2	1.3	1.5	0.8	1.6	2.4
	BMW17	0.05	0.2	1.7	1.9	0.8	1.2	2.0
	BMW18	0.09	0.4	2.8	3.3	0.8	1.8	2.6
	BMW19	0.06	0.3	1.9	2.2	0.3	2.5	2.8
	BMW20	0.11	0.6	4.0	4.2	0.8	1.6	2.4
	BPF21	0.04	0.2	1.3	1.5	0.7	0.8	1.6
	BPF22	0.02	0.1	0.6	0.7	0.6	1.3	1.9
	BPF23	0.03	0.1	1.0	1.1	0.8	0.7	1.5
	BPF24	0.03	0.1	0.9	1.0	0.6	1.0	1.6
	BPF25	0.03	0.2	1.1	1.2	0.5	0.9	1.4
	BPF26	0.02	0.1	0.8	0.9	0.9	0.8	1.8
	BPF27	0.01	0.1	0.3	0.4	0.6	0.5	1.1
	BPF28	0.03	0.1	0.8	0.9	0.4	0.5	0.9
	BPF29	0.04	0.2	1.2	1.4	0.7	0.9	1.6
	BPF30	0.10	0.5	3.5	4.0	0.8	1.0	1.8
	Average	0.05	0.2	1.7	1.9	0.7	1.2	1.9
	Min-Max	0.01-0.11	0.1-0.6	0.3-4.0	0.4-4.2	0.2-1.7	0.5-2.5	0.9-3.1

* Value obtained from radon concentration multiplied by the transfer coefficient (10^{-4}), BMW = Ban Mae Wan site, BPF = Ban Pang Fan site.

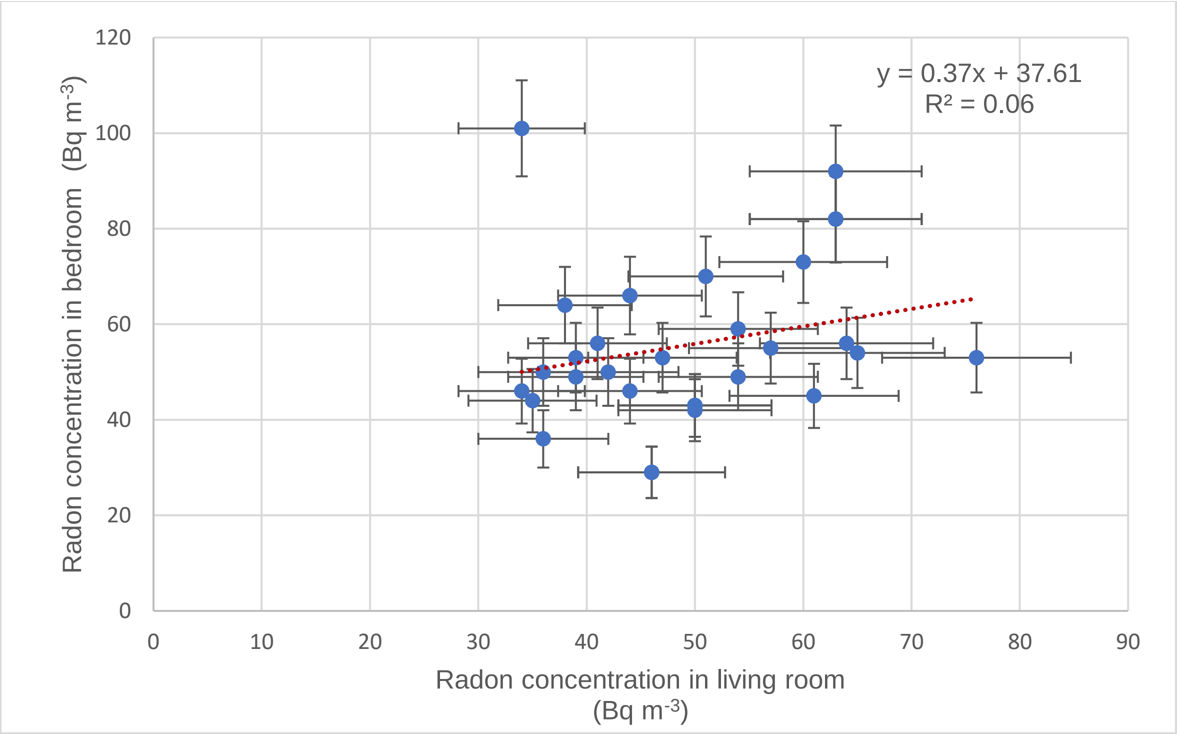

SUPPLEMENTARY FIGURE S1. Data points were plotted against radon concentration in the living rooms and the bedrooms with linear regression (red dotted line). $r^2 = 0.06$, $P>0.05$.

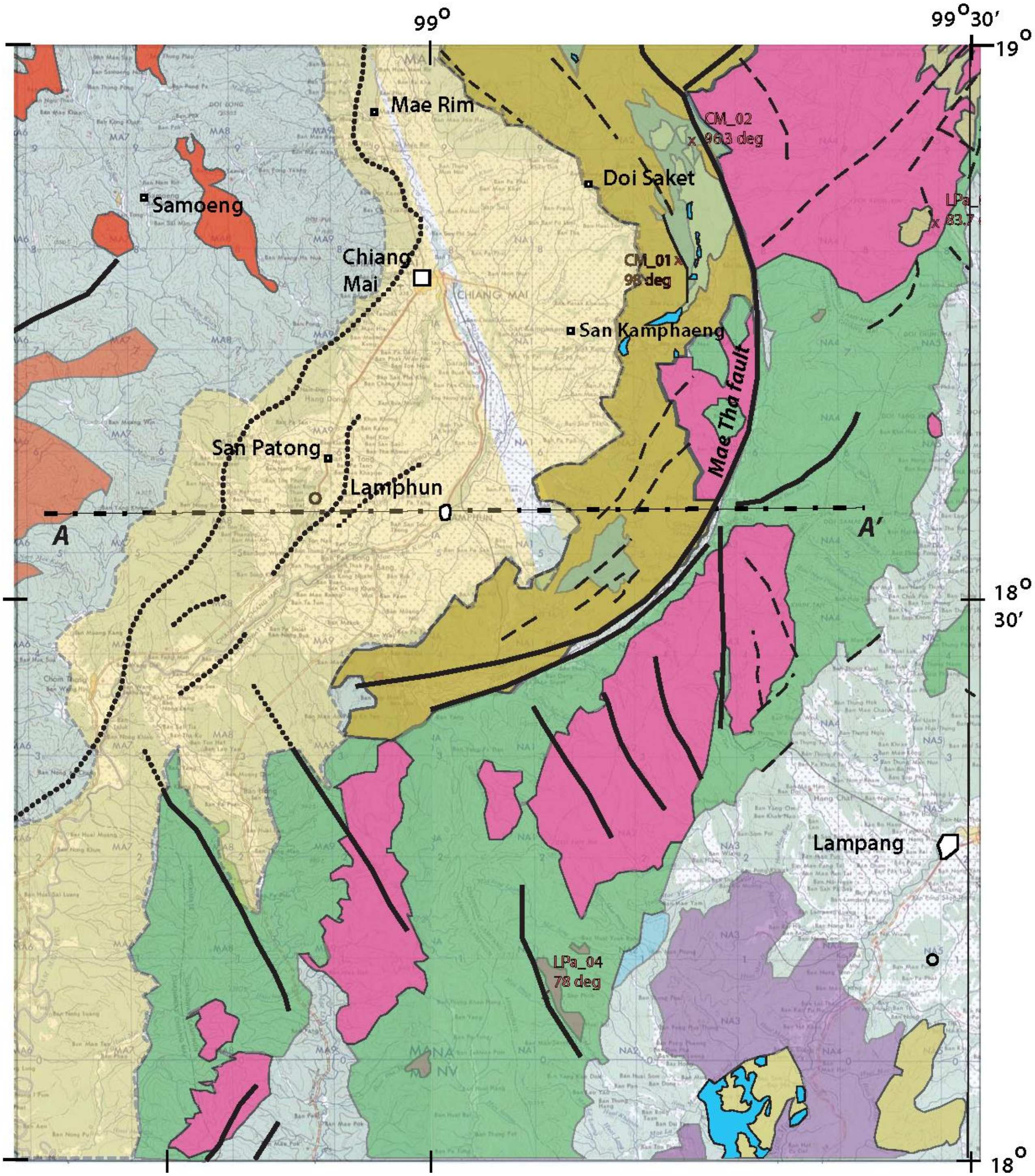

SUPPLEMENTARY FIGURE S2. Geologic map of the Chiang Mai basin.[29]

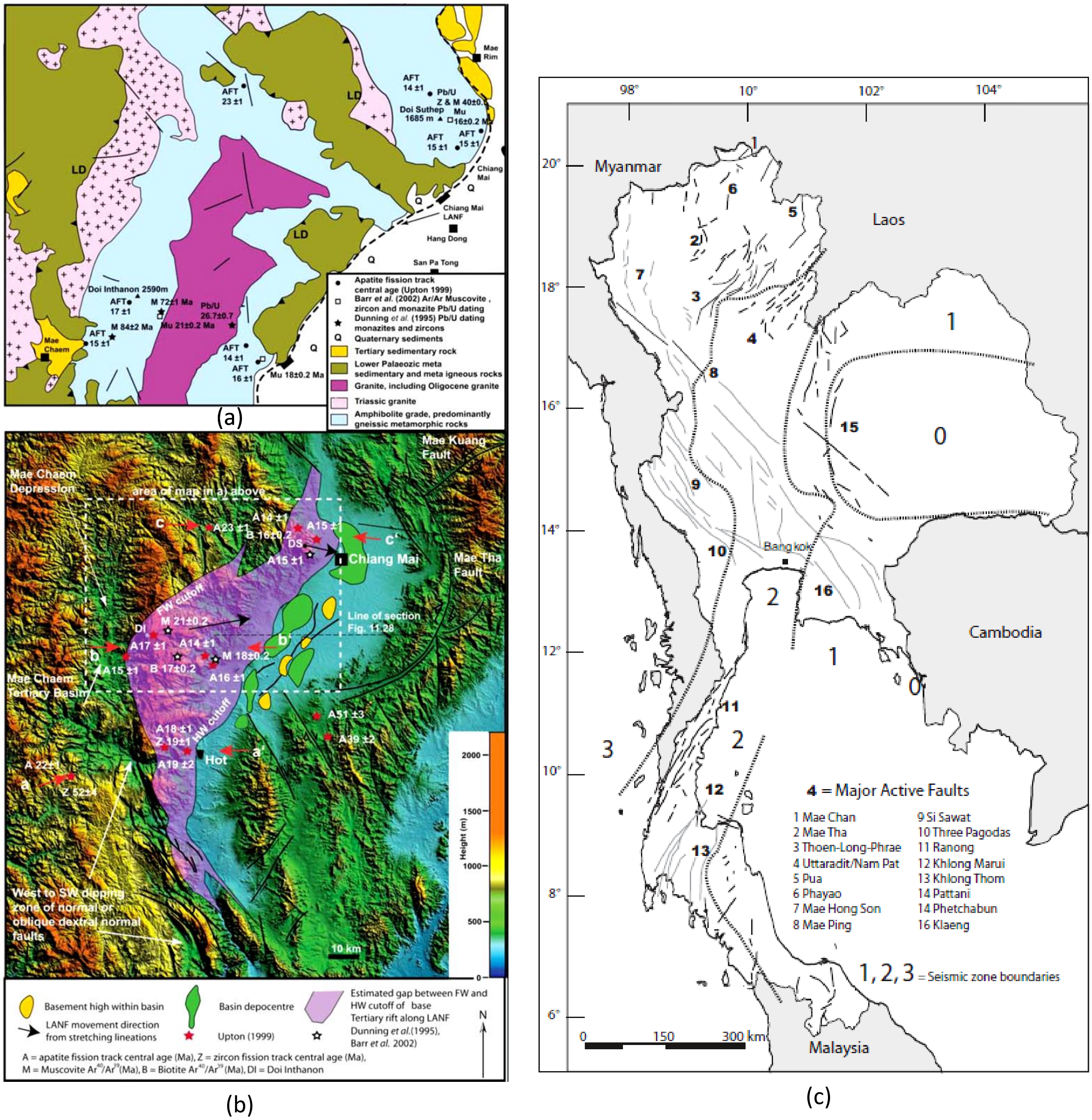

SUPPLEMENTARY FIGURE S3. The Mae Tha fault and active faults and seismic activity zones. (a) Detailed geological map of the west side of the Chiang Mai Basin (b) Map illustrating key regional features of the Chiang Mai Basin, (c) Map showing the active faults of Thailand and seismic activity zones.[28]
